# Association of meteorological factors with seasonal activity of influenza A subtypes and B lineages in subtropical western China

**DOI:** 10.1017/S0950268818003485

**Published:** 2019-03-04

**Authors:** M. Pan, H. P. Yang, J. Jian, Y. Kuang, J. N. Xu, T. S. Li, X. Zhou, W. L. Wu, Z. Zhao, C. Wang, W. Y. Li, M. Y. Li, S. S. He, L.L. Zhou

**Affiliations:** 1 Sichuan Center for Disease Control and Prevention, Chengdu 610041, China; 2 Guiyang Center for Disease Control and Prevention, Guiyang 550003, China; 3 West China School of Basic Medical Sciences & Forensic Medicine, Sichuan University, Chengdu 610041, China; 4 Panzhihua Center for Disease Control and Prevention, Panzhihua 617000, China; 5 Department of Medical Technology, West China School of Public Health, Sichuan University, Chengdu 610041, China

**Keywords:** Humidity, influenza A subtypes, influenza B lineages, meteorological factors, pandemic, seasonality, subtropical western China

## Abstract

The seasonality of individual influenza subtypes/lineages and the association of influenza epidemics with meteorological factors in the tropics/subtropics have not been well understood. The impact of the 2009 H1N1 pandemic on the prevalence of seasonal influenza virus remains to be explored. Using wavelet analysis, the periodicities of A/H3N2, seasonal A/H1N1, A/H1N1pdm09, Victoria and Yamagata were identified, respectively, in Panzhihua during 2006–2015. As a subtropical city in southwestern China, Panzhihua is the first industrial city in the upper reaches of the Yangtze River. The relationship between influenza epidemics and local climatic variables was examined based on regression models. The temporal distribution of influenza subtypes/lineages during the pre-pandemic (2006–2009), pandemic (2009) and post-pandemic (2010–2015) years was described and compared. A total of 6892 respiratory specimens were collected and 737 influenza viruses were isolated. A/H3N2 showed an annual cycle with a peak in summer–autumn, while A/H1N1pdm09, Victoria and Yamagata exhibited an annual cycle with a peak in winter–spring. Regression analyses demonstrated that relative humidity was positively associated with A/H3N2 activity while negatively associated with Victoria activity. Higher prevalence of A/H1N1pdm09 and Yamagata was driven by lower absolute humidity. The role of weather conditions in regulating influenza epidemics could be complicated since the diverse viral transmission modes and mechanism. Differences in seasonality and different associations with meteorological factors by influenza subtypes/lineages should be considered in epidemiological studies in the tropics/subtropics. The development of subtype- and lineage-specific prevention and control measures is of significant importance.

## Introduction

Type A and B influenza viruses have caused significant morbidity, mortality and economic burden worldwide [1, 2]. Influenza virus has an annual cycle, with a peak occurring in winter in temperate regions [3, 4]. However, seasonal activity of influenza virus in the tropics/subtropics has not been well characterised. The annual or semi-annual cycles, year-round activity, as well as periodicity transitions have been identified [4, 5].

Epidemiological analyses and experimental studies have indicated that cold temperature and low levels of absolute humidity (AH) are strongly associated with influenza epidemics in temperate regions [6–8]. In the tropics/subtropics, however, the climatic drivers of influenza epidemics are inconsistent. Increased seasonal activity of influenza virus was reported to be associated with higher humidity and/or precipitation in some areas, while no significant association or contradictory effects were observed in other locales [9–11].

Currently, influenza A virus subtype H3N2 (A/H3N2) and the 2009 pandemic influenza A virus subtype H1N1 (A/H1N1pdm09), as well as two influenza B lineages, B/Victoria/2/1987-like (Victoria) and B/Yamagata/16/1988-like (Yamagata), co-circulate in human population [12, 13]. A/H1N1pdm09 emerged in 2009 and caused the 2009 H1N1 pandemic. After the pandemic, A/H1N1pdm09 replaced the pre-existing seasonal influenza A virus subtype H1N1 (seasonal A/H1N1) [12–15]. The epidemic peaks of influenza type A and B have been reported to coincide less frequently in the tropics/subtropics than in temperate regions [16, 17]. However, epidemiological studies on the seasonality of individual influenza A subtypes and B lineages are limited; and the relationship between epidemics of individual subtypes/lineages and climate conditions is poorly understood. Moreover, it has been 9 years since the 2009 pandemic but the epidemiological characteristics of A/H1N1pdm09 during the post-pandemic period and the impact of the pandemic on the seasonal activity of the resident influenza subtypes/lineages remain to be explored.

The objectives of this study were to characterise and compare the temporal distribution of influenza subtypes/lineages during the pre-pandemic, pandemic and post-pandemic years, as well as to examine the relationships between influenza epidemics and meteorological factors in a subtropical city in detail. Since the diverse seasonal patterns of influenza virus in the tropics/subtropics, analysing surveillance data with greater spatial resolution (i.e. at the city level rather than on a national scale) is important for understanding viral global persistence and for developing local prevention and control measures. In this study, epidemiological and virologic data were collected during 2006–2015 in Panzhihua. Panzhihua is located in Sichuan Province in subtropical southwestern China. It covers an area of 7434 km^2^ and is the first industrial city in the upper reaches of the Yangtze River. The seasonality and distribution of A/H3N2, seasonal A/H1N1, A/H1N1pdm09, Victoria and Yamagata were characterised; and the relationship between seasonal activity of influenza virus and climate variables was assessed. We also calculated the frequency of influenza B vaccination mismatches.

## Materials and methods

### Influenza surveillance data

Epidemiological and virologic data during 2006–2015 in Panzhihua were obtained from the Chinese National Influenza-like Illness Surveillance Network (CNISN). Panzhihua is located at the 26.57°N and 101.67°E and is characterised by a monsoon-influenced humid subtropical climate [18], with modest annual temperature differences, concentrated precipitation, abundant sunshine and strong solar radiation. Influenza surveillance was conducted year-round based on a sentinel hospital-based influenza surveillance system, including two Grade III level A hospitals (top-class in China) in Panzhihua. In each hospital, 5–15 (⩾20 since 2013) nasopharyngeal swab specimens were collected each week from patients with influenza-like illness (ILI), who had not received antiviral treatment and within 3 days after the onset of symptoms. ILI was defined by the World Health Organization (WHO) as sudden onset of fever with measured body temperature ⩾38 °C and a cough or sore throat in the absence of other diagnoses. Madin–Darby canine kidney cells and/or specific pathogen-free chicken embryos were used to inoculate clinical specimens. The types, subtypes and lineages of influenza isolates were identified by haemagglutination inhibition (HI) test and/or real-time reverse transcription PCR (rRT-PCR) assay. During 2006–2008, virus culture and HI test were used to identify types, subtypes and lineages of influenza isolates. From 2009 to 2015, both virus culture and rRT-PCR assay were applied although the majority of specimens were identified by virus culture and HI test. A total of 83.0%, 40.2%, 98.0%, 96.4%, 79.4%, 99.3% and 91.5% of specimens tested in 2009, 2010, 2011, 2012, 2013, 2014 and 2015, respectively, were subtyped using virus culture and HI test. ILI consultation and influenza virologic surveillance were carried out based on the guideline for national influenza surveillance [19]. The primers and probes used for rRT-PCR are summarised in Table S1.

### Ethics statement

Influenza surveillance data used in this study was part of the CNISN. CNISN was supported by the National Health Commission of the People's Republic of China. Data were collected based on the guideline for national influenza surveillance [19] coordinated and supported by the Chinese Center for Disease Control and Prevention. Surveillance data were aggregated monthly and were analysed anonymously. Therefore, ethical approval was not required.

### Meteorological data

Meteorological data in Panzhihua during 2006–2015, including monthly mean temperature (°C), vapour pressure (VP, hPa) and relative humidity (RH, %), as well as total amount of precipitation (cm) and sunshine hours (h) were obtained from China Meteorological Administration [20]. VP was used as a measure of AH as some previous studies [8, 21].

### Influenza periodicity and seasonality analyses

Three pre-pandemic years (2006–2008), the pandemic year (2009) and six post-pandemic years (2010–2015) were included in the analyses. Unsubtyped influenza A virus-positive isolates were allocated to A/H3N2, seasonal A/H1N1 or A/H1N1pdm09, according to the seasonal ratio of each subtype. Influenza B virus-positive specimens that were not determined were allocated to Victoria, or Yamagata, according to the seasonal ratio of each lineage, excluding 2010 when 24 out of 33 (72.3%) influenza B virus-positive specimens were not determined. Because the A/H1N1pdm09 did not emerge in Panzhihua until October 2009, the annual ratio of 2009 was separately calculated for the first 9 months and the remaining 3 months. Time series of the monthly influenza-positive rates which were calculated as monthly numbers of positive specimens (all influenza viruses, or each A subtype, or each B lineage) divided by monthly total numbers of specimens tested were compiled.

Wavelet analysis which has been used previously for various infectious diseases data [22, 23] was applied to characterise seasonality. Wavelet analysis is able to identify periodicities in time-series data and help track variations in periodicity over time [24]. The sowas package [25] implemented in MATLAB R2016b (Mathworks, Natick, MA, USA) was applied to conduct wavelet analysis. To take into account the increase in the number of specimens and the 2009 H1N1 pandemic, time series were square root-transformed prior to analysis, according to previous studies [26–28].

### Statistical analyses of virologic and meteorological data

A type or subtype/lineage was considered ‘dominant’ in Panzhihua if it accounts for 50% or more of the total isolates for a particular year. A type or subtype/lineage was considered ‘codominant’ if it accounts for between 40% and 50% of the annual isolates. To assess the differences in subtype/lineage distribution, the positive rates of a given subtype/lineage were compared during pre-pandemic, pandemic and post-pandemic years using *χ*^2^ or Fisher's exact tests as appropriate. Two-sided *P* values were reported and a *P* value satisfying *P* < 0.05 was considered as statistically significant. The updated influenza vaccine based on recommendations from the WHO is used in Panzhihua from September of one year to August of the following year. Accordingly, a vaccination year was defined as the period from September of one year to August of the following year. Since the surveillance period in this study is from 2006 to 2015, only nine vaccination years were included.

Correlations of seasonal activity of influenza subtypes/lineages with monthly mean temperature, VP and RH, as well as monthly total amount of precipitation and sunshine hours were evaluated using Spearman's rank correlation. Stepwise multiple linear regression models were also used to avoid possible collinearity. In order to evaluate the relationship between A/H1N1pdm09 activity and meteorological factors after the pandemic, the pandemic year 2009 was excluded. Statistical analyses were performed using SPSS v21 (SPSS Inc., Chicago, IL, USA).

## Results

### Seasonality and periodicity of influenza epidemics in Panzhihua

**Fig. 1.**
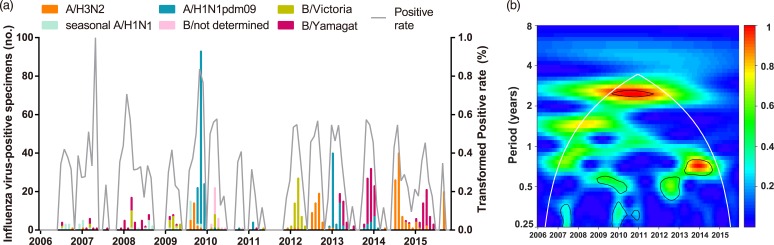
Composite influenza virus activity in Panzhihua during 2006–2015. (a) The monthly positive rate of all influenza viruses combined and the monthly positive numbers of laboratory-confirmed A/H3N2, seasonal A/H1N1, A/H1N1pdm09, Victoria and Yamagata cases. (b) Wavelet power spectrum of the monthly activity of all influenza viruses combined. Black lines highlight periodicities that reach statistical significance of 95% based on 1000 Monte Carlo simulation. The region outside the white-curved cone indicates the presence of edge effects. The power values were shown in the panel on the right. Time series have been square-root transformed.

A total of 6892 respiratory specimens were collected and influenza viruses were isolated from 737 (10.69%) specimens during 2006–2015 (Table 1). The composite influenza virus activity including all laboratory-confirmed influenza viruses exhibited non-stationary periodicity (Fig. 1). Semi-annual cycles (*P* < 0.05), with one peak occurring in March–April and the other in September or November, were observed during the 2009 H1N1 pandemic and 2012. Semi-annual and annual cycles were not identified during the other surveillance years.

**Table 1.** Number, positive rates and proportions of influenza virus by season in Panzhihua, 2006–2015

			No. (frequency^a^) of influenza A subtypes and B lineages							Proportion^b^ of influenza A subtypes and B lineages						
Year	Specimens, No.	Positive specimens, No. (frequency)	A	A/H3N2	Seasonal A/H1N1	A/H1N1pdm	B	Victoria	Yamagata	A	A/H3N2	Seasonal A/H1N1	A/H1N1pdm	B	Victoria	Yamagata
2006^c^	208	16 (7.69%)	11 (5.29%)	1 (0.48%)	10 (4.81%)	NA	3 (1.44%)	1 (0.48%)	2 (0.96%)	68.75%	6.25%	62.50%	NA	18.75%	6.25%	23.81%
2007	170	16 (9.41%)	6 (3.53%)	1 (0.59%)	5 (2.94%)	NA	10 (5.88%)	2 (1.18%)	8 (4.71%)	37.50%	6.25%	31.25%	NA	62.50%	12.50%	24.71%
2008	471	45 (9.55%)	10 (2.12%)	0 (0.00%)	10 (2.12%)	NA	35 (7.43%)	9 (1.91%)	26 (5.52%)	22.22%	0.00%	22.22%	NA	77.78%	20.00%	4.65%
2009^c^	1144	189 (16.52%)	170 (14.86%)	22 (1.92%)	15 (1.31%)	133 (11.63%)	19 (1.66%)	15 (1.31%)	4 (0.35%)	89.95%	11.64%	7.94%	70.37%	10.05%	7.94%	0.42%
2010^d^	256	35 (13.67%)	2 (0.78%)	0 (0.00%)	NA	2 (0.78%)	33 (12.89%)	9 (3.52%)	0 (0.00%)	5.71%	0.00%	NA	5.71%	94.29%	25.71%	15.69%
2011	493	7 (1.42%)	5 (1.01%)	1 (0.20%)	NA	4 (0.81%)	2 (0.41%)	1 (0.20%)	1 (0.2%)	71.43%	14.29%	NA	57.14%	28.57%	14.29%	1.45%
2012	633	100 (15.80%)	50 (7.90%)	49 (7.74%)	NA	1 (0.16%)	50 (7.90%)	50 (7.90%)	0 (0.00%)	50.00%	49.00%	NA	1.00%	50.00%	50.00%	3.23%
2013	1076	145 (13.48%)	67 (6.23%)	1 (0.09%)	NA	66 (6.13%)	78 (7.25%)	0 (0.00%)	78 (7.25%)	46.21%	0.69%	NA	45.52%	53.79%	0.00%	27.27%
2014	1106	108 (9.76%)	90 (8.14%)	82 (7.41%)	NA	8 (0.72%)	18 (1.63%)	0 (0.00%)	18 (1.63%)	83.33%	75.93%	NA	7.41%	16.67%	0.00%	39.20%
2015	1335	76 (5.69%)	27 (2.02%)	27 (2.02%)	NA	0 (0.00%)	49 (3.67%)	0 (0.00%)	49 (3.67%)	35.53%	35.53%	NA	0.00%	64.47%	0.00%	10.84%
Total	6892	737 (10.69%)	438 (6.36%)	184 (2.67%)	40 (0.58%)	214 (3.11%)	297 (4.31%)	87 (1.26%)	186 (2.70%)	59.43%	24.97%	5.43%	29.04%	40.30%	11.80%	25.24%
Differences of influenza virus-positive rates between the years before, during and after the 2009 H1N1 pandemic																
Pre-pandemic	849	77 (9.07%)	27 (3.18%)	2 (0.24%)	25 (2.94%)	NA	48 (5.65%)	12 (1.41%)	36 (4.24%)							
Pandemic	1144	189 (16.52%)	170 (14.86%)	22 (1.92%)	15 (1.31%)	133 (11.63%)	19 (1.66%)	15 (1.31%)	4 (0.35%)							
Post-pandemic	4899	471 (9.61%)	241 (4.92%)	160 (3.27%)	NA	81 (1.65%)	230 (4.69%)	60 (1.22%)	146 (2.98%)							
*χ*^2^ (*P*-value)																
Pre- *vs*. post-pandemic		0.618	*0.02649	*0.000	NA	NA	0.229	0.648	0.053							
Pre- *vs*. pandemic		*0.000	*0.000	*0.000637	*0.010133	NA	*0.000	0.845	*0.000							
Post- *vs*. pandemic		*0.000	*0.000	*0.016719	NA	*0.000	*0.000	0.812	*0.000							

NA, not available.

* Results considered to be statistically significant (*P* < 0.05).

a The positive rate of laboratory-confirmed influenza cases among all specimens collected.

b The proportion of subtype- or lineage-positive cases among all laboratory-confirmed influenza cases.

c A total of two isolates were of mixed infections.

d A total of 24 influenza B isolates in 2010 were not characterised into lineages.

**Fig. 2.**
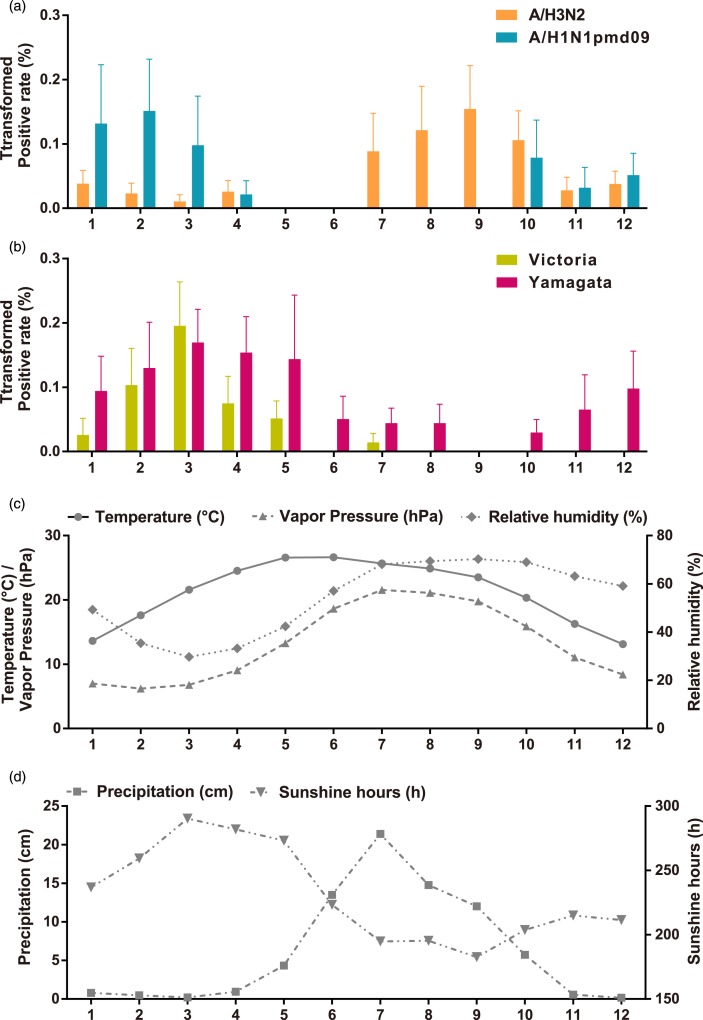
Monthly average influenza virus activity and climatic parameters in Panzhihua during 2006–2015. (a) The monthly average positive rates (square-root transformed) of influenza A subtypes. Error bars show standard errors based on variation between years. (b) The same as A, but for influenza B lineages. (c) Temperature, vapour pressure, and relative humidity. (d) Precipitation and sunshine hours.

To describe the seasonality of each subtype/lineage in Panzhihua, 184, 40, 214, 87 and 186 influenza cases of A/H3N2, seasonal A/H1N1, A/H1N1pdm09, Victoria and Yamagata were analysed (Table 1). Individual subtypes and lineages generally demonstrated the annual or semi-annual cycles. During 2006–2015, A/H3N2 exhibited an annual cycle with a peak in August–September. Wavelet analysis showed that the annual cycle was statistically significant (*P* < 0.05) during 2012–2015 (Fig. 2a and Fig. S1). Both annual and semi-annual cycles were observed for seasonal A/H1N1 during 2006–2009 and this virus was not detected after 2009 (Fig. S1). A/H1N1pdm09 emerged in Panzhihua in October 2009 and demonstrated an annual cycle (*P* < 0.05) with a peak in November (Fig. S1) 2009. During the post-pandemic years, A/H1N1pdm09 generally demonstrated an annual cycle with a peak occurring in January–February, which was statistically significant (*P* < 0.05) during 2013–2014 (Fig. 2a and Fig. S1). Victoria lineage exhibited an annual cycle (*P* < 0.05) with a peak in March in most surveillance years (Fig. 2b and Fig. S1). Yamagata lineage was also characterised by an annual peak but could occur in November–April during the surveillance years (Fig. 2b and Fig. S1).

### Association of influenza epidemics with climatic parameters

The monthly statistics for the climate variables in Panzhihua during 2006–2015 were summarised in Table S2. The monthly averages of these climatic variables over the study period were shown in Figure 2. Both annual mean sunshine hours and solar radiation occupy the forefront of China [29]. The average temperature is highest in May–June (average 26.6 °C) and is lowest in December (average 13.1 °C). RH is lower in February–April (29.7–35.4%) and higher in July–November (63.2–70.3%). AH, as measured by VP, tends to be lower in February–March (6.2–7.8 hPa) and higher in July–August (21.1–21.6 hPa). Much of the rainfall occurred in June–September, with the average monthly total precipitation around 13.5–21.4 cm. The average monthly total hours of sunshine ranges from 182.6 to 290.3 h.

The relationship between climate variables and the activity of influenza subtypes/lineages was evaluated on a monthly basis. Table 2 shows the Spearman's rank correlation coefficients between the monthly influenza virus prevalence data and meteorological factors. A/H3N2 activity was positively associated with RH, while A/H1N1pdm09 activity was negatively associated with temperature, precipitation and VP. Higher level of sunshine hours and lower level of RH and VP was associated with increased prevalence of both Victoria and Yamagata. Additionally, Victoria activity was negatively associated with precipitation. Stepwise multiple linear regression analyses showed that higher A/H3N2 activity was only associated with higher RH (coefficient = 0.234, *P* = 0.001, adjusted *R*^2^ = 0.081), while A/H1N1pdm09 prevalence was only negatively correlated with VP (coefficient = −0.740, *P* = 0.002, adjusted *R*^2^ = 0.118). Higher Victoria activity was driven by lower RH (coefficient = −0.344, *P* = 0.000, adjusted *R*^2^ = 0.239), while higher Yamagata activity was promoted by lower VP (coefficient = −0.663, *P* = 0.013, adjusted *R*^2^ = 0.044).

**Table 2.** Spearman's rank correlation between meteorological factors and influenza activity in Panzhihua, 2006–2015

	Spearman's rank correlation				
	Temperature (°C)	Vapour pressure (hPa)	Relative humidity (%)	Precipitation (cm)	Sunshine hours (h)
A/H3N2	−0.101404	0.121189	0.220769*	0.078295	−0.052152
A/H1N1pdm09^a^	−0.371049**	−0.430149***	−0.220043	−0.363226**	0.142835
Victoria	0.075987	−0.324126***	−0.441326***	−0.282564**	0.427359***
Yamagata	−0.109817	−0.216964*	−0.238596**	−0.110855	0.220669*

*P < 0.05, **P < 0.01, ***P < 0.001.

a Analysis excluded the 2009 H1N1 pandemic year to focus on the prevalence pattern of A/H1N1pdm09 during inter-pandemic years.

### Subtype/lineage distributions of influenza epidemics

Overall, 59.4% and 40.3% of influenza-positive cases during the 10-year surveillance were type A and B, respectively (Table 1). Compared with those in the pre-pandemic period, statistically significant (*P* < 0.05) decrease of positive rates in the pandemic was observed for seasonal A/H1N1 and Yamagata, while an increase of positive rate was seen for A/H3N2. Similar positive rates between pre-pandemic and pandemic years were observed for Victoria. After the pandemic, seasonal A/H1N1 was not detected in Panzhihua and the positive rate of A/H1N1pdm09 became significantly lower than in the pandemic (Table 1). There was not any difference in positive rates of Victoria and Yamagata between the post-pandemic and the pre-pandemic seasons, while the positive rates of A/H3N2 in the post-pandemic years were significantly higher than in the pre-pandemic period. The predominant influenza subtypes/lineages observed in Panzhihua are shown in Figure 1a and Table 1. There were at least two subtypes/lineages co-circulating each year. One of influenza A subtypes or one of B lineages predominated in most years, except in 2012 and 2013 when an influenza A subtype and a B lineage were codominant. Influenza A subtypes generally co-circulated in most years, although A/H3N2 were not detected in 2008 and 2010 and A/H1N1pdm09 were not detected in 2015. Two influenza B lineages co-circulated in many years, except in 2013–2015 when Victoria were not detected and in 2010 and 2012 when Yamagata were not detected.

**Fig. 3.**
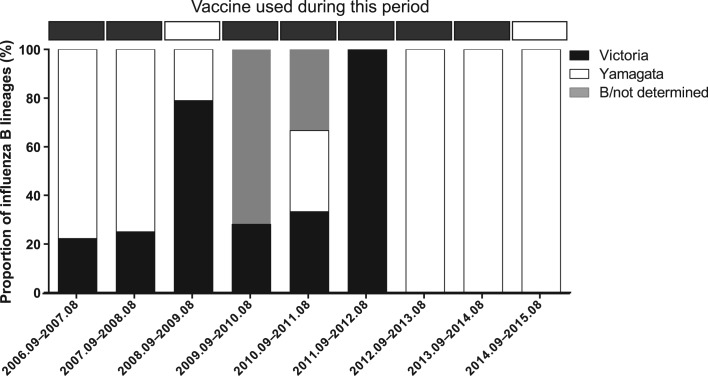
Proportion of influenza B lineages in Panzhihua by vaccination year (September 2006–August 2015). Black boxes represent Victoria, white boxes represent Yamagata and grey boxes represent influenza B isolates that were not determined. Horizontal bars on the top show the WHO-recommended influenza B vaccine lineage.

So far, only trivalent inactivated influenza vaccine (TIV) have been available in the market in China. It was shown that the influenza B strain included in TIV did not match the dominant circulating lineage at least in five of the nine vaccination years studied (Fig. 3).

## Discussion

Ten-year surveillance demonstrated non-stationary seasonality of composite influenza virus activity in Panzhihua. Although well-defined seasonal patterns, such as the annual or semi-annual cycles were observed in many subtropical regions, cities with non-stationary periodicity were also reported [4, 5, 30]. However, the seasonality of individual influenza subtypes/lineages and the seasonal drivers remain to be investigated.

Our study found that the seasonal patterns among influenza subtypes/lineages were not the same and the associations with meteorological factors were different by subtype/lineage. A/H3N2 generally demonstrated an annual cycle with a peak in summer–autumn. An annual cycle with a peak mainly occurring in summer was observed for A/H3N2 during 2004–2013 in Hong Kong [31]. We further found that A/H3N2 activity was positively associated with RH in Panzhihua. This association was partly consistent with previous epidemiological analyses in eastern and southern China, which found that RH was positively correlated with influenza A activity [10, 21, 32]. However, lower RH was also found to be associated with higher influenza A activity in subtropical Japan [11]. The periodicity of seasonal A/H1N1 was non-stationary during the pre-pandemic and pandemic years. Additional surveillance data might be necessary to identify the epidemic pattern and climatic predictors of seasonal A/H1N1. A/H1N1pdm09 replaced seasonal A/H1N1 after the pandemic and exhibited an annual cycle with a peak in winter. Statistical analyses indicated that higher A/H1N1pdm09 activity was driven by lower AH. This relationship was also shown by previous analyses of A/H1N1pdm09 in temperate and subtropical/tropical regions [7, 33]. Influenza B lineages showed an annual cycle, respectively, as previous studies of both influenza B lineages combined [30]. Our study found that the peak month of Victoria mainly occurred in March while the peak month of Yamagata would occur during November–April. Regression analyses demonstrated that seasonal activity of Victoria was inversely associated with RH while Yamagata activity was negatively associated with AH. The inverse association between influenza B activity and humidity was different from previous studies in some subtropical cities, likes Hong Kong and Okinawa, which showed that the epidemics of all influenza B viruses combined could be promoted by higher RH/AH [10, 11]. Animal experiments demonstrated that transmission of both influenza B lineages could be enhanced at colder temperature [34]. But the impact of humidity on influenza B transmission remains to be examined.

Differences in seasonality might be driven by several factors, including viral survival and transmission, host susceptibility and behaviour, and the environment. Our findings suggested that the different effects of weather conditions on influenza subtypes/lineages might be related to the regional heterogeneity of influenza seasonality. Animal experiments showed that aerosol transmission of type A and B influenza strains was highly effective at low temperature (5 °C) [34–36]. This temperature condition might be common in temperate region. However, Panzhihua is a warm subtropical city, with limited annual temperature differences. Over the 10-year surveillance, the lowest average monthly temperature was 13.1–13.6 °C in winter and the highest was 26.5–26.6 °C in late spring and early summer. Therefore, the association of temperature with influenza epidemics might not be significant. Our analyses showed that the associations between humidity and influenza activity varied by subtype/lineage in Panzhihua. This might indicate that at the context of a subtropical city, each influenza subtype/lineage could have a most suitable humidity with different temperature condition. RH might perform effects on influenza virus transmission at different level [35, 36]. Aerosol transmission of influenza A could be maximal at low RH (20% and 35%) while minimal at high RH (80%). Notably, effective transmission (75% or 100%) of type A influenza virus was also observed at 20 °C and 65% RH. The peak months of A/H3N2 mainly occurred in August–September coinciding with the peak months of RH in Panzhihua. In August–September, the average monthly temperature was 23.5–22.6 °C and average monthly RH was 69.4–70.3%, which might support the active transmission of A/H3N2. While lower AH/RH in winter–spring might be more suitable for virus activity of A/H1N1pdm09, Victoria and Yamagata. Previous epidemiological analyses, for example, in Hong Kong and Suzhou, showed that higher RH was correlated with higher influenza A activity [10, 21, 32]. However, these studies did not analyze influenza A subtypes or influenza B lineages separately. In addition, RH is a measure of the water content in a gas, relative to the maximum capacity of that gas to hold water vapour at a given temperature. RH reflects human's feeling of humidity while AH measures the mass of water vapour per unit volume of a gas. It is suggested that aerosol transmission of influenza virus shows a direct dependence on AH, rather than varying with both RH and temperature [6–8]. However, several transmission results based on RH and temperature could not be explained by AH alone [35, 36]. It is possible that humidity (either RH or AH) and temperature affect transmission of influenza subtypes/lineages by more than one mechanism.

It is suggested that novel antigenic variants might interrupt the circulation of the resident viruses, possibly through competition for susceptible hosts [37]. We found some statistically significant changes in positive rates for individual subtypes/lineages during the pandemic and post-pandemic years. However, periodicity changes were not detected for influenza virus in Panzhihua, which was identified in Chengdu in southwestern China (unpublished data). The interaction between the novel pandemic strain and other resident influenza viruses, virus virulence variation and host susceptibility might be related to the changes in positive rates of influenza subtypes/lineages.

Understanding the seasonal pattern and the climatic drivers of influenza virus is of significant importance for predicting epidemic timing and optimizing interventions. Influenza vaccine has not been included in the national immunisation programme in China and the average national vaccination coverage was just around 1.5–2.2% between 2004 and 2014 [38]. There is a growing interest in establishing routine immunisation programmes nationwide [39]. Differences in seasonality by subtype/lineage indicated that except for the winter influenza epidemics, the summer–autumn peak of A/H3N2 should also be considered in vaccination campaign in Panzhihua. Moreover, since the influenza B strain included in TIV did not match the dominant circulating strain in five of the nine vaccination years, it is suggested to use quadrivalent influenza vaccine (QIV) to provide broad protection against all circulating lineages. Our study found some statistically significant changes in positive rates for individual subtypes/lineages since the 2009 H1N1 pandemic. Therefore, year-round influenza surveillance is needed to be strengthened to monitor on-going changes.

There are some limitations in this study. First, our data focused only on patients with ILI from sentinel hospitals. A random sample of people with influenza-like symptoms from both hospitals and the community might be more appropriate. Second, the relationship between seasonal activity of influenza virus and some other seasonal drivers, such as virus interaction, host susceptibility, social behaviour and so on, remains to be verified. Third, virus culture and rRT-PCR assay might have different sensitivity of influenza virus detection [40]. However, the subtype/lineage distribution and seasonality are unlikely to be changed [41]. Our wavelet analysis results showed that the periodicity of influenza subtypes/lineages were consistent during 2006–2015 in Panzhihua, except for seasonal A/H1N1 which were not detected after the 2009 H1N1 pandemic.

This study expands our knowledge regarding the epidemiological characteristics of individual influenza subtypes/lineages as well as the association with meteorological factors in the subtropics. In summary, we found differences in seasonality and different associations with climatic variables by subtypes/lineages. These findings revealed that the role of weather conditions in regulating virus activity could be complicated which might be related to the diverse transmission modes and mechanism of influenza virus. Studies of influenza seasonality and its relationship with climatic drivers should therefore account for individual subtypes/lineages. Moreover, it is necessary to increase year-round influenza surveillance and develop subtype/lineage-specific prevention and control measures. In addition, QIV is recommended to provide protection against both influenza B lineages.

## Supplementary material

For supplementary material accompanying this paper visit https://doi.org/10.1017/S0950268818003485. click here to view supplementary material
