# Supplementary material for: Association of meteorological factors with seasonal activity of influenza A subtypes and B lineages in subtropical western China
Source: Epidemiol Infect. 2019 Mar 4;147:e72. doi: 10.1017/S0950268818003485 (PMC6518542; doi:10.1017/S0950268818003485)
Supplement: Supplementary file 1 [file S0950268818003485sup001.docx]

Epidemiology and Infection

**Association of meteorological factors with seasonal activity of influenza A subtypes and B lineages in subtropical western China**

M. Pan^1,†^, H.P. Yang^1,†^, J. Jian^2^, Y. Kuang^3^, J.N. Xu^1^, T.S. Li^1^, X. Zhou^4^, W.L. Wu^4^, Z. Zhao^3^, C. Wang^5^, W.Y. Li^3^, M.Y. Li^3^, S.S. He^1^, L.L. Zhou^3,*^

^1^Sichuan Center for Disease Control and Prevention, Chengdu 610041 China

^2^Guiyang Center for Disease Control and Prevention, Guiyang 550003 China

^3^West China School of Basic Medical Sciences & Forensic Medicine, Sichuan University, Chengdu 610041 China

^4^Panzhihua Center for Disease Control and Prevention, Panzhihua 617000 China

^5^Department of Medical Technology, West China School of Public Health, Sichuan University, Chengdu 610041 China

^†^ These authors contributed equally to this work.

^*^ Correspondence: Linlin Zhou

West China School of Basic Medical Sciences & Forensic Medicine, Sichuan University, No.17 People's South Road, Chengdu, 610041, Sichuan, P.R. China

Email: zhoulinlin@scu.edu.cn

**Supplementary Material**

**Table S2.** The primers and probes used for real-time reverse transcription PCR (rRT-PCR) for determining the types, subtypes, and lineages of influenza isolates.

| **Primer/Probe** | **Base composition** |
| --- | --- |
| FluA-Forward | 5’-GACCRATCCTGTCACCTCTGAC-3’ |
| FluA-Reverse | 5’-GGGCATTYTGGACAAAKCGTCTACG-3’ |
| FluA-probe | 5’-TGCAGTCCTCGCTCACTGGGCACG-3’ |
| FluB-Forward | 5’-TCCTCAACTCACTCTTCGAGCG-3’ |
| FluB-Reverse | 5’-CGGTGCTCTTGACCAAATTGG-3’ |
| FluB-probe | 5’-CCAATTCGAGCAGCTGAAACTGCGGTG-3’ |
| BHA- Forward | 5’-AGACCAGAGGGAAACTATGCCC-3’ |
| BHA- Reverse | 5’-TCCGGATGTAACAGGTCTGACTT-3’ |
| VIC- probe | 5’-CAGACCAAAATGCACGGGGAAHATACC-3’ |
| YAM- probe | 5’-CAGRCCAATGTGTGTGGGGAYCACACC-3’ |
| CNICH3-Forward | 5’-ACCCTCAGTGTGATGGCTTTCAAA-3’ |
| CNICH3-Reverse | 5’-TAAGGGAGGCATAATCCGGCACAT-3’ |
| CNICH3-Probe | 5’-ACGAAGCAAAGCCTACAGCAACTGT-3’ |
| pdmH1Forward | 5’-GGGTAGCCCCATTGCAT-3’ |
| pdmH1Reverse | 5’-AGAGTGATTCACACTCTGGATTTC-3’ |
| pdmH1Probe | 5’-TGGGTAAATGTAACATTGCTGGCTGG-3’ |
| H1-F247 | 5’-AACATGTTACCCAGGGCATTTCGC-3’ |
| H1-R361 | 5’-GTGGTTGGGCCATGAGCTTTCTTT-3’ |
| H1-P278 | 5’-GAGGAACTGAGGGAGCAATTGAGTTCAG-3’ |

**Table S2.** Summary of monthly statistics for climatic variables in Panzhihua, 2006–2015

| **Climatic variable** | **Mean** | **S.D.** | **Median** | **Minimum** | **Maximum** |
| --- | --- | --- | --- | --- | --- |
| **Temperature (°C)** | 21.2 | 4.9 | 22.3 | 11.9 | 29.6 |
| **Vapor pressure (hPa)** | 13.2 | 5.7 | 12.1 | 4.8 | 22.9 |
| **Relative humidity (%)** | 53.9 | 15.6 | 59.0 | 24.0 | 77.0 |
| **Precipitation (cm)** | 6.2 | 7.9 | 1.8 | 0.0 | 35.2 |
| **Sunshine hours (h)** | 230.6 | 48.4 | 237.3 | 120.3 | 328.2 |

**Figure S1.** Seasonal prevalence and wavelet power spectrum of influenza A subtypes and B lineages in Panzhihua, 2006–2015. (A) The monthly positive rates of laboratory confirmed A/H3N2, seasonal A/H1N1, A/H1N1pdm09, Victoria, and Yamagata. (B) Wavelet power spectrum of the monthly activity of influenza virus. White lines highlight periodicities that reach statistical significance of 95%. The region outside the white-curved cone indicates the presence of edge effects. The power values were shown in the panel on the right. Time series have been square-root transformed.
